# Epigenomic assessment of differential pathological and clinical presentation in the Lewy body dementias

**DOI:** 10.1002/alz.088852

**Published:** 2025-01-09

**Authors:** Joshua Harvey, Jennifer Imm, Byron Creese, Leonidas Chouliaras, Emma Dempster, Clive G Ballard, John T O'Brien, Dag Aarsland, Jonathan Mill, Lasse Pihlström, Ehsan Pishva, Katie Lunnon

**Affiliations:** ^1^ University of Exeter, Exeter, Devon UK; ^2^ University of Cambridge, Cambridge UK; ^3^ Department of Old Age Psychiatry, Kings College, London UK; ^4^ Oslo University Hospital, Oslo Norway; ^5^ Maastricht University, Maastricht Netherlands

## Abstract

**Background:**

The Lewy body dementias are a class of neurodegenerative disease encompassing Parkinson’s disease dementia (PDD) and Dementia with Lewy bodies (DLB). Despite similar clinical and neuropathological profiles, the principal differentiating feature is the sequence in which symptoms develop. A question still remains as to a biological basis of this difference and a potential explanation is epigenetic mechanisms, molecular processes which alter the way in which the underlying genetic sequence is expressed.

**Method:**

DNA methylation was quantified using the Illumina EPIC array for 932 samples from the anterior cingulate cortex (ACC, n = 462) and the prefrontal cortex (PFC, n = 470), sourced from 481 unique donors. Samples were defined based on antemortem records as neurological controls (n = 103), PD (n = 123), PDD (n = 101), DLB (n = 95) and Alzheimer’s Disease (AD, n = 59). Weighted gene correlation network analysis (WGCNA) was conducted, controlling for confounders to define co‐methylated networks with evidence of association to pathological (Braak NFT and LB) outcomes and clinical subgroups, including presentation of neuropsychiatric symptoms. Significantly associated modules were tested using gene ontology analysis (GO, missMethyl) and expression enrichment in publically available single nucleus RNA sequencing datasets (Expression Weighted Cell Type Enrichment Analysis, EWCE).

**Result:**

11 co‐methylation modules (ACC = 9, PFC = 2) showed FDR corrected significant association to either a pathological or clinical outcome. The most significantly associated module was observed in the ACC (Figure A & B), with association with both Braak LB stage (P = 1.49 x 10^‐5^) and PDD presentation stratification (P = 1.41 x 10^‐6^). The module showed significant GO term enrichment relevant to inflammation (Figure C) and EWCE analysis confirmed expression of genes annotated to this module were significantly enriched in microglial cell types. The module contained epigenetic loci annotated to C1QB, PLA2G15 and ERGIC1, all of which showed nominal clinical groupwise association in a clinical EWAS analysis of the same cohort.

**Conclusion:**

Epigenetic alterations show evidence of insight into brain region specific pathological processes with differential stratification between DLB and PDD. Ongoing analysis in this cohort looks to resolve sex specific stratified analyses.

